# Epicure: a European epidemiological study of patients with an advanced or metastatic Urothelial Carcinoma (UC) having progressed to a platinum-based chemotherapy

**DOI:** 10.1186/s12885-016-2782-3

**Published:** 2016-09-23

**Authors:** N. Houédé, G. Locker, C. Lucas, H. Soto Parra, U. Basso, D. Spaeth, R. Tambaro, L. Basterretxea, F. Morelli, C. Theodore, L. Lusuardi, N. Lainez, A. Guillot, G. Tonini, J. Bielle, X. Garcia Del Muro

**Affiliations:** 1 Institut de Cancérologie du Gard - CHU Caremeau, 30029 Nîmes, Cedex 9 France; 2 Department of Internal Medicine I, Währinger Gürtel.18-20, 1090 Vienne, Austria; 3 Institut de Recherche Pierre Fabre, 45 place Abel Gance, 92100 Boulogne-Billancourt, France; 4 Oncologia Medica, P.O Gaspare Rodolico, Via Santa Sofia 78, 95123 Catania, Italy; 5 Istituto Oncologico Veneto IOV-IRCCS Oncologia Medica, 1 Via Gattamelata 64, Padova, Italy; 6 Centre d’Oncologie de Gentilly, 2 rue Marie Marvingt, 54100 Nancy, France; 7 Istituto Nazionale Tumori IRCCS Fondazione Pascale, Via Mariano Semmola, 80131 Napoli, Italy; 8 Hospital Universitario Donostia, Begiristain Doktorea Pasealekua 117-20080, Donostia, Gipuzkoa - San Sebastián Spain; 9 Fondazione Casa Sollievo della Sofferenza Oncologia, Viale Cappuccini 1, San Giovanni Rotondo, Foggia Italy; 10 Hopital Foch, 40 rue Worth, 92150 Suresnes, France; 11 Reparto di Urologia - Ospedale di Bressanone, Via Dante 51, 39042 Bressanone, Italy; 12 Hospital de Navarra - Virgen del Camino, Oncología Médica, Calle de Irunlarrea, 4 planta baja, 31008 Pamplona, Spain; 13 Institut de cancérologie de la Loire, 108 bis avenue Albert Raimond, 42271 Saint Priest en Jarez, Cedex France; 14 Policlinico Universitario Campus Bio-medico Oncologia Medica, Via Alvaro del Portillo 200, 00128 Roma, Italy; 15 ICO L’Hospitalet, Avinguda Granvia, 199-203, 08907 L’Hospitalet de Llobregat, Barcelona Spain

**Keywords:** Urothelial carcinoma, Bladder cancer, Cisplatinum, Vinflunine, Epidemiology, Practice, Second-line, Metastatic

## Abstract

**Background:**

Platinum-based systemic chemotherapy is considered the backbone for management of advanced urothelial carcinomas. However there is a lack of real world data on the use of such chemotherapy regimens, on patient profiles and on management after treatment failure.

**Methods:**

Fifty-one randomly selected physicians from 4 European countries registered 218 consecutive patients in progression or relapse following a first platinum-based chemotherapy. Patient characteristics, tumor history and treatment regimens, as well as the considerations of physicians on the management of urothelial carcinoma were recorded.

**Results:**

A systemic platinum-based regimen had been administered as the initial chemotherapy in 216 patients: 15 in the neoadjuvant setting, 61 in adjuvant therapy conditions, 137 in first-line advanced setting and 3 in other conditions. Of these patients, 76 (35 %) were initially considered as cisplatin-unfit, mainly because of renal impairment (52 patients). After platinum failure, renal impairment was observed in 44 % of patients, ECOG Performance Status ≥ 2 in 17 %, hemoglobinemia < 10 g/dL in 16 %, hepatic metastases in 13 %. 80 % of these patients received further anticancer therapy. Immediately after failure of adjuvant/neoadjuvant chemotherapy, most subsequent anticancer treatments were chemotherapy doublets (35/58), whereas after therapy failure in the advanced setting most patients receiving further anticancer drugs were treated with a single agent (80/114). After first progression to chemotherapy, treatment decisions were mainly driven by Performance Status and prior response to chemotherapy (>30 % patients). The most frequent all-settings second anticancer therapy regimen was vinflunine (70 % of single-agent and 42 % of all subsequent treatments), the main reasons evoked by physicians (>1 out of 4) being survival benefit, safety and phase III evidence.

**Conclusion:**

In this daily practice experience, a majority of patients with urothelial carcinoma previously treated with a platinum-based therapy received a second chemotherapy regimen, most often a single agent after an initial chemotherapy in the advanced setting and preferably a cytotoxic combination after a neoadjuvant or adjuvant chemotherapy. Performance Status and prior response to chemotherapy were the main drivers of further treatment decisions.

## Background

More than 90 % of all cancers of the urinary tract are transitional cell carcinomas of the urothelium (urothelial carcinoma UC), 90 % being localized in the bladder [1, 2]. UC is a major health problem. In the European Union, bladder cancer is the fifth most frequently diagnosed malignant tumor with more than 124,000 new cases in 2012 corresponding to 4.7 % of all human neoplasms. It accounts for about 41,000 deaths in Europe [3].

Those patients with muscle-invasive UC are at high risk of recurrence or progression, and half of them relapse after radical surgery. The majority of relapses are distant metastases and 10–15 % of patients are already metastatic at diagnosis [4]. Metastatic UC is an aggressive disease with a median survival not exceeding 6 months if untreated [5]. Chemotherapy plays an important role in the treatment of advanced stages of the disease. For first-line treatment of advanced or metastatic UC, a cisplatin-containing combination chemotherapy is considered the standard, either the classical MVAC (methotrexate, vinblastine, adriamycin, cisplatin) regimen or dose-dense MVAC and gemcitabine-cisplatin regimens which are better tolerated [6, 7]. The median survival is 13–15 months with these regimens in the cisplatin-eligible patients [2, 6, 8]. However, up to 50 % of patients are not eligible for a first-line cisplatin-containing chemotherapy because of their poor performance status (PS) and/or comorbidities. For these patients, there is no clear standard treatment but a carboplatin-based regimen or a single agent therapy are considered acceptable alternatives, according to European guidelines [2, 8, 9].

A cisplatin-based neoadjuvant treatment is also recommended by clinical guidelines [2, 8, 10] for some high-risk patients, before radical cystectomy. The role of adjuvant chemotherapy is more controversial but a meta-analysis of nine randomized trials and a large observational study suggested Disease-Free Survival and Overall Survival (OS) benefits for the patients who received cisplatin-based adjuvant chemotherapy [11, 12].

Second-line phase II data are highly variable with results depending on patient selection. Response rates for treatment of relapse with mono-chemotherapy are lower than those with combinations, but Progression-Free Survival and OS remain short with both options. In addition, prognostic factors in second-line were only recently established [13], making difficult the interpretation of oldest study results.

After platinum-based chemotherapy failure, the only chemotherapeutic agent approved in Europe is vinflunine. Some physicians also consider of re-challenging cisplatin-sensitive patients if progression occurs at least 6–12 months after first-line cisplatin-based combination. Both treatment modalities are endorsed by clinical guidelines (EAU, ESMO, ASCO) together with inclusion in clinical trials [2, 8, 10].

Nevertheless, not all patients can benefit from second-line therapy after they have progressed to a first platinum-based chemotherapy. Probable reasons are the drug prescription limitations, impaired general health status that allows only best supportive care because of potential adverse effects. In some cases, non-approved drugs are used, based on physicians’ experience.

Most UCs are diagnosed at the superficial stage and it is more complex to collect information on patients diagnosed with an advanced or metastatic stage, which explains why information on patient profiles and disease management is very limited at time of second systemic treatment.

After decades of unmet medical need with no strong evidence-based results and no specifically approved drug, physicians treatment decision may vary a lot. In addition there is no precise guidance according to the patient profile and prognostic factors.

Thus, there is a need to better characterize these patients and clarify physicians’ practices. This could lead to optimizing the use of available treatments.

The objective of this non-interventional study is to define the characteristics of patients when progression (resistance or relapse) is demonstrated after a first systemic platinum-based treatment and to report the physician’s therapeutic attitudes both in theory from physician’s perspective and in daily practice according to the actual characteristics of patients attending a consultation during the survey period.

## Methods

### Study design

The study was a European ambispective survey reporting epidemiology and practices in the management of urothelial carcinoma (UC), following progression to a platinum-based chemotherapy given in adjuvant, neoadjuvant or metastatic settings.

The study aimed to draw an accurate picture of the current practices. So it was formally requested that usual medical practices should not be impacted by the study process.

A total sample of 280 patients was planned from approximately 70 centers selected at random and located in the participating countries: Austria, France, Italy and Spain.

The lists of centers in each country were established on the basis the centers had physicians experienced in the management of advanced or metastatic UC (≥6 patients/year).

**Fig. 1:**
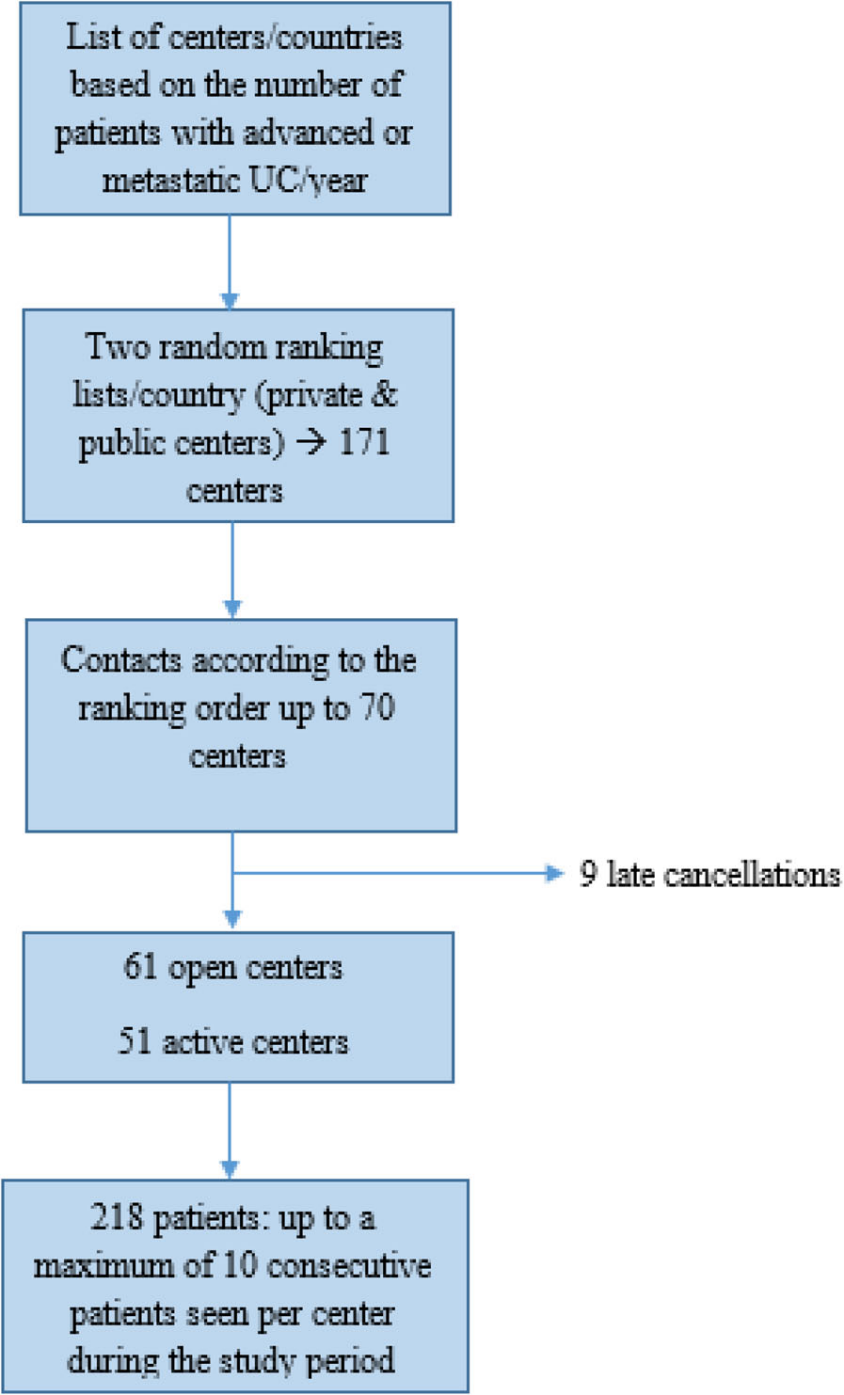
Flowchart of Center Selection and Patient Recruitment

The random lists of centers took into account the private and public status of the institutions in accordance with each country mode of management for the disease at this stage. This led to a list of 171 physicians within the four participating countries. Sixty-one out of 70 planned centers finally participated due to 9 late cancellations, and 51 centers actively recruited patients (Fig. 1).

All patients signed a specific informed consent if requested or at least received detailed written information. In compliance with the regulations of each participating country, the study was approved by national authorities as a non-interventional study and assessed by ethics review boards of each participating institution, wherever applicable.

Two types of information were collected:

- Firstly, real-life patient data from case report forms. Data were collected on the first series of consecutive patients seen on a visit, with an expected number of 4 to 8 patients per center, up to a maximum of 10 patients in a given center during the study period. Registered patients had to fit the inclusion criteria: age over 18 years, locally advanced or metastatic UC, pre-treatment with a platinum-based chemotherapy (regardless of its setting: neoadjuvant chemotherapy, adjuvant chemotherapy or palliative first-line in advanced/metastatic disease), having shown progression to the platinum-based treatment. Patients having received prior platinum-free systemic chemotherapy only were excluded.
  Information collected included: initial patient characteristics and prior treatments, patient characteristics and comorbidities at the time of progression, disease management in the post-platinum setting.
- Secondly, a questionnaire was filled by all participating physicians regarding their practices, at the time of patient inclusion. Physicians were asked how in theory he/she should manage the patient (anticancer treatment or alternatives) according to the patient characteristics after one systemic platinum-based chemotherapy regimen.

Statistics were mainly descriptive. Continuous data were summarized using the following items: frequency, median, range, mean, standard deviation and standard error if relevant. Categorical data were presented in contingency tables with frequencies and percentages of each modality (including missing data modality). 95 % confidence intervals were calculated following the exact method. Furthermore, the relationship between the type of therapy (monochemotherapy or combination) received after progression and patient profiles after failure of first platinum treatment, was assessed by both univariate and multivariate analyses. In these exploratory analyses, a threshold of *p* < 0.05 was considered for indicating a significant impact of patient characteristics.

## Results

Two hundred and eighteen patients were included in the study by 51 active centers between April 2013 and April 2014. The recruited patients were 104 in Italy, 54 in Spain, 35 in France and 25 in Austria.

### Centers and patients characteristics

The 51 active centers were located in Austria (*n* = 7), France (*n* = 7), Italy (*n* = 21) and Spain (*n* = 16). The split between public and private practice was 7/0 in Austria, 6/1 in France, 16/5 in Italy and 15/1 in Spain. Among the 218 patients under study, 51 were followed in private centers, and 167 in public centers.

The mean number of patients recruited per center was 4.3 (between 1 and 10). Thirty-four centers recruited up to 4 patients, 11 centers between 5 and 9 patients, and 6 centers recruited 10 patients.

Of the 218 patients, 5 were excluded from the analysis because 2 did not received any platinum-based chemotherapy and 3 had multiple different consecutive chemotherapy regimens. However, these patients were included in the patient characteristics analysis.

Males represented 84 % of the patients and median age was 68. Thirty-three patients (15.1 %) were ≥ 75 years old. Regarding the number of systemic chemotherapy treatments at study entry, 45 patients (21 %) had received just one previous chemotherapy regimen; 136 (62 %) had received 2 regimens, and 37 (17 %) had received 3 or more regimens.

At registration, the treatment status of the patients was: ongoing chemotherapy *n* = 140 (64 %), best supportive care *n* = 42 (19 %), pending decision *n* = 28 (13 %) and other situations (i.e. remission period, palliative surgery) *n* = 8 (4 %).

Disease location at diagnosis was the bladder for 166 patients (76 %), upper urinary tract for 40 patients (18 %), and urethra or other/multiple locations for 12 patients (6 %). The stages at diagnosis comprised non muscle-invasive tumors for 24 patients (11 %), muscle-invasive for 62 patients (28 %) and locally advanced or metastatic disease for 132 patients (61 %). In this latter group, 49 patients (22 %) had distant metastases at diagnosis.

Most patients (*n* = 171 – 78 %) were initially treated with surgery including radical cystectomy, partial cystectomy or nephro-ureterectomy. Only 8 patients (4 %) were treated by radiotherapy.

At the time of first platinum chemotherapy, 76 patients (35 %) were considered unfit for cisplatin, whereas 142 patients (65 %) were fit enough to receive a cisplatin-based chemotherapy. Table 1 displays the reasons for considering patients as unfit for cisplatin (some patients may have had several reasons).

**Table 1:** Conditions contributing to cisplatin ineligibility

Reason(s) for cisplatin-ineligibility *n* = 76	
Single reason	**66 (87 %)**
Renal impairment only	44 (58 %)
PS ≥ 2 only	9 (12 %)
Single reason not specified	7 (9 %)
Heart failure only	5 (7 %)
Hearing impairment only	1 (1 %)
Multiple reasons	**10 (13 %)**
PS ≥ 2 + renal impairment	5 (7 %)
PS ≥ 2 + heart failure	1 (1 %)
PS ≥ 2 + other	1 (1 %)
Renal impairment + Hearing impairment	1 (4 %)
Renal impairment + other	1 (1 %)
Renal impairment + Hearing impairment + other	1 (1 %)

Other reasons included age or comorbidities

As first systemic chemotherapy, 123 (56 %) patients received a cisplatin-based regimen and 93 (43 %) patients a carboplatin-based regimen. Two patients were treated with a platinum-free regimen. Of the 213 patients who could be analyzed according to the setting of their first systemic chemotherapy regimen, 76 patients received their platinum therapy for neoadjuvant (15 patients) or adjuvant (61 patients) therapy objectives. Among them, approximately one third (26 patients) was treated with carboplatin and 50 patients with cisplatin. Regarding the remaining 137 patients who received first-line treatment for advanced disease, 66 were administered carboplatin and 71 cisplatin-based regimen. 45 % of patients (*n* **=** 61) displayed objective response, of whom one third (*n* **=** 20) had complete response. 27 % (*n* **=** 37) had disease stabilization and 26 % (*n* **=** 36) had progressive disease. At the time of subsequent post-platinum treatment decision, following treatment failure, many patients had poor general conditions (Table 2). Renal impairment was observed in 44 % of patients, ECOG PS ≥ 2 in 17 %, hemoglobinemia <10 g/dl in 16 %, hepatic metastases in 13 %.

**Table 2:** Patient profile at time of post-platinum treatment decision

Patient profile at time of post-platinum treatment decision (number of available patients)	*N*	%
Age (*n* = 218)		
*≥ 75 years*	33	15
ECOG PS (*n* = 213)		
*PS 0/1*	76/101	36/47
*PS ≥ 2*	36	17
Renal impairment (*n* = 217)		
*Creatinine clearance < 60 mL/min*	95	44
*Creatinine clearance < 40 mL/min*	25	12
Low hemoglobin value (*n* = 217)		
< 10 g/dL	35	16
Neutropenia (or leucopenia) (*n* = 217)	6	3
Hepatic metastases (*n* = 216)	28	13
Hepatic impairment (*n* = 217)	6	3
Clinically relevant cardiac toxicity (*n* = 217)	13	6

Only 63 (29 %) patients were considered as not having any major constraint or co-morbid condition at the time of subsequent treatment decision.

Despite their condition, most of the patients (*n* = 175 – 80 %) received further chemotherapy, 71 receiving combination therapy and 104 monotherapy. Only 18 patients (8 %) were managed by best supportive care. The remaining patients were waiting for treatment decisions or managed by other options. The chemotherapy regimens used after neoadjuvant/adjuvant platinum treatments were most often (60 %) a combination therapy, preferentially including a platinum agent. The main drug combined with platinum was gemcitabine. On the other hand, after platinum therapy given in the advanced setting, the majority of patients (70 %) were treated with single-agent chemotherapy. Vinflunine represented 42 % of all subsequent chemotherapy regimens and 70 % of single agent therapy. Taxanes were the second type of chemotherapy used, representing 22 % of single agent therapy with paclitaxel being used 3.6 times more often than docetaxel.

The different therapeutic options chosen are summarized in Table 3.

**Table 3:** Disease management immediately following failure of the first platinum-based chemotherapy regimen

Initial platinum-based chemotherapy *n*, (%)	Neo/adjuvant setting, *n* = 76	Advanced setting, *n* = 137
Cisplatin-based	50 (66 %)	71 (52 %)
Carboplatin-based	26 (34 %)	66 (48 %)
Subsequent chemotherapy *n*, (%)	**58 (76 %)**	**114 (83 %)**
Single agent	**23**	**80**
*Vinflunine/Taxanes*	*15/5*	*57/18*
*Gemcitabine/Other agent*	*2/1*	*2/3*
Combination therapy	**35**	**34**
*cisplatin-based*	*11*	*5*
*carboplatin-based*	*14*	*22*
*Other*	*10*	*7*
Subsequent management by BSC n, (%)	**7 (9 %)**	**11 (8 %)**
Pending decision or other^a^ n, (%)	**11 (14 %)**	**12 (9 %)**

^a^Other situations: scheduled surgery, radiotherapy or chemotherapy

In the descriptive analysis, the main patient characteristics impacting the choice of any possible second-line treatment were performance status (50 %), response to previous chemotherapy (poor response: 33 %; good response: 22 %), age (23 %), renal impairment (23 %), multiple comorbidities (7 %) and visceral metastases (7 %).

Additional univariate and multivariate analyses looked into the association between patient characteristics at the time of progression to the first systemic chemotherapy and the subsequent treatment (single agent therapy or polychemotherapy regimen). The explored patient parameters were the established prognostic factors in second line (hemoglobinemia < 10 g/dL; ECOG PS; liver metastases) and other characteristics considered to potentially impact on treatment decision (age <75 or ≥ 75; renal function – creatinine clearance below or over 60 mL/min; presence or not of cardiac toxicities; response to prior systemic chemotherapy; regimen of initial chemotherapy (cisplatin-based or not); number of cycles; reason of discontinuation of initial chemotherapy; time to progression after initial systemic chemotherapy and major toxicities during the course of prior cytotoxic regimen). In univariate analysis, 4 factors were significantly associated with either single agent or combination therapy: hemoglobin level (*p* = 0.0034), response to initial chemotherapy (*p* < 0.0001), reason for discontinuation (*p* = 0.002) and time to progression (*p* < 0.0001). The multivariate analysis confirmed the association with response to previous chemotherapy (*p* = 0.0356) and time to progression (*p* = 0.0063), clearly impacting the choice of subsequent treatment; hemoglobin level was at the limit of significance (*p* = 0.0553).

### Usual practices for first systemic chemotherapy (according to physician’s questionnaire)

For the participating physicians (*n* = 51), the most commonly firstly used chemotherapy regimen is a doublet of platinum plus gemcitabine, whether patients are fit or not to receive cisplatin.

Tables 4 and 5 depict the preferred choices of physicians for first systemic chemotherapy, in patients fit and unfit for cisplatin, according to the setting (neoadjuvant-adjuvant or palliative for advanced or metastatic disease).

**Table 4:** Preferred choices of physicians for first systemic chemotherapy, in patients eligible for cisplatin

Usual physician chemotherapy regimen for 1st systemic anti-cancer therapy in patients eligible to cisplatin	Set of Physicians *N* = 51
As neoadjuvant or adjuvant chemotherapy^a^	
Missing	-
MVAC	3 (5.9 %)
HD-MVAC	1 (2.0 %)
GEM-cisplatin	42 (82.4 %)
GEM-cisplatin or (HD)-MVAC	3 (5.9 %)
Other	3 (5.9 %)
As palliative first-line chemotherapy	
Missing	-
MVAC	2 (3.9 %)
HD-MVAC	-
GEM-cisplatin	46 (90.2 %)
GEM-cisplatin or (HD)-MVAC	2 (3.9 %)
Other	1 (2.0 %)

^a^ One physician ticked two answers

**Table 5:** Preferred choices of physicians for first systemic chemotherapy, in patients not eligible for cisplatin

Usual physician chemotherapy regimen for 1st line anticancer systemic therapy in patients ineligible to receive cisplatin	Set of Physicians *N* = 51
As neoadjuvant or adjuvant chemotherapy	
Missing	-
Single agent	**-**
Chemotherapy doublet	**43 (84.3 %)**
Gemcitabine-carboplatin	40 (78.4 %)
Paclitaxel-carboplatin	1 (2.0 %)
Docetaxel-carboplatin	1 (2.0 %)
Gemcitabine-Paclitaxel	1 (2.0 %)
Other	**1 (2.0 %)**
None	7 (13.7 %)
As palliative first-line chemotherapy	
Missing	-
Single agent	**2 (3.9 %)**
Gemcitabine	2 (3.9 %)
Carboplatin (carboplatin)	-
Chemotherapy doublet	**49 (96.1 %)**
Gemcitabine-carboplatin	44 (86.3 %)
Paclitaxel-carboplatin	2 (3.9 %)
Docetaxel-carboplatin	1 (2.0 %)
Gemcitabine-Paclitaxel	1 (2.0 %)
Other	1 (2.0 %)

### Factors impacting treatment decisions following progression or relapse to a first platinum-based therapy (according to physician’s questionnaire)

Most physicians (42 out of 51, 82 %) declared that the way they would theoretically manage the disease after a progression or relapse does not really differ whether the first systemic chemotherapy was administered in the perioperative setting or as palliative first-line treatment**.**

The factors most impacting treatment decisions following progression or relapse to a first platinum-based therapy are performance status (for 90 %), comorbidities (for 55 %), response to prior chemotherapy (for 43 %), renal impairment (for 35 %) and progression-free interval after prior chemotherapy (for 31 %). European guidelines, patients/families requests and drug access also impact choices but for only 14, 10 and 8 % of the clinicians, respectively.

Best supportive care is not considered in patients with PS 0–1 but only as possible option by 26 % of physicians in case of PS ≥ 2 without associated renal impairment and by 45 % in case of combined adverse conditions. A vast majority of physicians consider in theory a single-agent therapy in post-platinum setting whatever the PS is (72–82 % of cases, except for patients having combined PS ≥ 2 and impaired renal function for whom physicians balance treatment decision with best supportive care −51–45 %). Only in patients with PS 0 and normal renal function, a doublet is considered by 31 % of physicians, with no standard regimen but gemcitabine-cisplatin in more than a half of them.

Vinflunine is the most frequent treatment option in patients with PS 0 or 1 independently of renal function (34–38 physicians out of the set of 51: 67–75 %), but is rarely perceived as a possible treatment in case of PS 2 (<8 %). The main reasons claimed by physicians for using vinflunine (Table 6) are phase 3 study evidence (67 %), safety profile (41 %), survival benefit (29 %) and vinflunine European approval (26 %).

**Table 6:** Reason(s) for choosing vinflunine for management of a patient following progression/relapse to an initial platinum-based chemotherapy

Reason(s) for choosing vinflunine for management of a patient after progression/relapse to an initial platinum-based chemotherapy, *n* = 51 physicians *(0 up to a maximum of 3 reasons could be given)*	
Phase III evidence	34 (66.7 %)
Safety profile	21 (41.2 %)
Survival benefit	15 (29.4 %)
Drug approval	13 (25.5 %)
Guidelines	12 (23.5 %)
Progression free survival	11 (21.6 %)
Convenience of administration	9 (17.6 %)
Good prior experience	6 (11.8 %)
Best efficacy expectations	5 (9.8 %)
Symptoms control	4 (7.8 %)
Quality of life	3 (5.9 %)
Simple scheme	3 (5.9 %)
Handable schedule	2 (3.9 %)
Disease stabilization rate	1 (2.0 %)
Patient/family request/other reason	-

The second most frequent option is paclitaxel single agent: 22–31 % of physicians, regardless of PS. Gemcitabine single agent is considered mainly in patients with PS ≥ 2 but only by 4–12 % of physicians in patients with PS 0–1.

## Discussion

This observational study analyzes the clinical practice in four European countries, reporting disease management of advanced UC. It describes the proportion and main characteristics of patients receiving a second systemic anticancer treatment without the patient selection biases related to drug clinical trials where patients are usually included with good PS and few comorbidities. It is the first survey assessing European routine medical practices in advanced stages of UC previously treated with a platinum-based chemotherapy. Two retrospective epidemiological studies were previously communicated as abstracts. One assessed the type of platinum treatment given in 298 patients with stage IV disease [14]. The second retrospective data collection was conducted in selected centers with the aim of assessing prognostic factors of OS [15]. None provided such detailed information on both first-line, subsequent treatments and patients characteristics in daily practice. This is of interest considering the current gaps in clinical guidelines, in particular for the management of patients with ECOG PS ≥ 2 or with comorbidities. In addition, there is no strong phase III scientific evidence supporting the few combination therapy options that are used in practice while there are no recommendations based on recently established prognostic factors. As a consequence, practices vary.

Three out of the four chosen countries for this study (France, Italy and Spain) are among the 5 major countries for incidence and mortality from bladder cancer in the European Union, the others being Germany and the United Kingdom [3]. In order to have centers representative of actual care of advanced UC, the number of centers contacted per country was proportional to the published country bladder cancer-related mortality.

The proportion of recruited patients closely mirrored the incidence and mortality of bladder cancer in the four participating countries [3] with slight variations: Austrian patients number was above expectations and French patients were less represented.

Possible selection biases have been limited through the inclusion of all consecutive patients in each center, over a maximum time corresponding to the study duration. However it is possible that patients in very poor health conditions, not able to attend an oncologic/urologic visit, were not included in the study.

This study provides important insights on the type of UC patients who receive platinum treatments in Europe. A majority of patients (63 %) received a first platinum-based chemotherapy regimen as palliative treatment in the advanced or metastatic stage.

At the time of first systemic chemotherapy decision, 65 % of the patients were theoretically fit to receive cisplatin but only 56 % of them received a cisplatin-based therapy. The contra-indications to cisplatin treatment are well-known but their respective frequencies in this population of patients have never been reported. In this survey, it was observed that 70 % of cisplatin-unfit patients had a single adverse condition for cisplatin use, either renal impairment (58 %) or a PS ≥ 2 (12 %), while hearing impairment was considered as single or combined reason in only 5 %.

The observed response rate in the survey (45 %) mirrors the expected response rates of 46 % with the MVAC regimen and 49 % with the gemcitabine-cisplatin regimen [16] but is quite important considering the rate of patients who did not receive a cisplatin-based regimen (43 %).

In the picture taken at registration, 80 % of patients receive a second anticancer systemic treatment. The post-platinum therapy was most often a single agent after chemotherapy administered in the advanced/metastatic setting, and preferably a cytotoxic combination after a neoadjuvant or adjuvant chemotherapy regimen. PS and prior response to chemotherapy were the main parameters that influenced treatment decisions (both >50 %) after a platinum-based therapy. The other published second-line prognosis factors (hemoglobinemia, liver metastasis) [13] were considered as impacting the treatment choice in less than 7 % of cases. When considering the univariate and multivariate analyses testing the association between patient characteristics and the decision of subsequent single agent or polychemotherapy regimen, the strongest association was found with results achieved with the first treatment (objective response and time to progression). Age (< or ≥ 75) did not impact on the choice of treating patients with single-agent or combination. Surprisingly, PS did not appear as an influencing factor; this may be due to the limited number of patients with PS ≥ 2 and/or to the fact that most patients treated by combination therapy or by vinflunine are PS 0 or 1.

The most frequent chemotherapy regimen after platinum was single agent vinflunine (42 % of all second anticancer systemic treatments). Taxanes (mainly paclitaxel) are still used as single agent but represent only 13 % of post-platinum chemotherapy treatments.

As of today, and outside of clinical trials, single agent therapy remains a standard in second-line treatment; actually chemotherapy doublets did not demonstrate extended survival rates even though they had shown higher response rates in phase II studies [17]. Regimens varied widely in cases where a combination therapy was administered in post-platinum setting, carboplatin being used quite often.

The real-life conditions of this study show that the health status and prognosis of patients seen in routine practice, are not worse than those of patients participating to the pivotal phase III vinflunine trial [18], in which PS 2 patients were excluded. Patients with PS 0-1- ≥ 2 were 36 %-47 %-17 % here vs 28 %-72 %-0 % in the phase III trial. Renal impairment (as defined by a creatinine clearance < 60 mL/min) was 44 % vs 47 %. The population in this survey showed relatively low visceral metastases involvement, with only 13 % patients presenting hepatic metastases (vs 29 % in the pivotal phase III). Another adverse prognosis factor, hemoglobinemia < 10 g/dL was reported in only 16 % of patients in this study as compared to 86 % in the phase III trial.

After platinum-based chemotherapy failure, vinflunine is the only chemotherapeutic agent approved in Europe. The approval was based on a randomized phase III trial investigating vinflunine plus best supportive care (BSC) versus BSC alone [18]. The results showed clinical benefit with a favorable safety profile and a survival benefit in favor of vinflunine, which was statistically significant in the eligible patient population, with a 22 % reduction of the risk of death being achieved [19].

Recently, different prospective or retrospective studies of vinflunine have been published, on the basis of German, Spanish, Greek and British series of patients receiving a second-line treatment or more [20–23]. In these studies patients with PS 2 represented 8–23 % and liver metastasis was reported in 17–29 % of patients. Interesting response rates were obtained, comprised between 13 and 29 % and overall survival was between 7.7 and 11.9 months.

Second-line response rates obtained with taxanes, ifosfamide, topotecan, pemetrexed and different tyrosine kinase inhibitors have ranged between 0 and 28 % in small phase II trials [24].

Gemcitabine displayed also interesting response rates in second-line treatment, but most patients already receive this drug in first-line [25]. Paclitaxel/gemcitabine studies have shown increased response rates, but no randomized phase III trial with an adequate comparator arm has been conducted to assess the true value and OS benefit of this second-line combination [5, 26].

Checkpoint inhibitors, e.g. targeting the PD-1/PD-L1 axis, hold promising potential with good tolerability in advanced UC [27]. Atezolizumab was recently approved in the United States by the Food and Drug Administration [28] on the basis of a large phase II study involving 310 patients with UC progressing following platinum treatment [29]. This approval was granted in a country where there was no approved drug in the second-line setting. In Europe, no checkpoint inhibitors have been approved yet for the treatment of UC. Combining chemotherapies with immunotherapies may provide valuable options with improved response rates and tolerable toxicity.

## Conclusion

This study conducted in four European countries reflects daily practice in the treatment of patients with urothelial carcinoma eligible for a first platinum-based chemotherapy either in adjuvant/neoadjuvant or in advanced/metastatic setting. It fills a knowledge gap on the characteristics of UC patients treated with platinum agents and on the reasons and modalities of further treatments. Cisplatin was used in 56 % of patients. Cisplatin-ineligibility appeared mainly due to renal dysfunction (68 %) and PS ≥ 2 (21 %).

A second chemotherapy regimen was administered in 80 % of patients. Most often this was a single agent following an initial systemic chemotherapy administered in the first-line advanced setting (70 % of patients); after a neoadjuvant or adjuvant chemotherapy, the preference was for a cytotoxic combination (60 % of patients). PS and prior response to chemotherapy were the main parameters that influenced disease management. The most frequent second systemic anticancer therapy was single agent vinflunine (42 % of all subsequent systemic therapies), the main reasons evoked by physicians being survival benefit, safety and phase III evidence.

## Abbreviations

ASCO: American Society of Clinical Oncology
BSC: Best supportive care
CI: Confidence interval
EAU: European Association of Urology
ECOG: Eastern Cooperative Oncology Group
ESMO: European Society of Medical Oncology
GEM: Gemcitabine
HD-MVAC: High-dose-intensity methotrexate, vinblastine, doxorubicin and cisplatin
HR: Hazard ratio
MVAC: Methotrexate, vinblastine, doxorubicin and cisplatin
OS: Overall survival
PD-1: Programmed death receptor 1
PD-L1: Programmed death-ligand 1
PS: Performance status
UC: Urothelial carcinoma
